# The World Health Organization Antenatal CorTicosteroids for Improving Outcomes in preterm Newborns (ACTION-III) Trial: study protocol for a multi-country, multi-centre, double-blind, three-arm, placebo-controlled, individually randomized trial of antenatal corticosteroids for women at high probability of late preterm birth in hospitals in low- resource countries

**DOI:** 10.1186/s13063-024-07941-0

**Published:** 2024-04-12

**Authors:** WHO ACTION Trials Collaborators, Temitope Adesiji Adegboyega, Ebunoluwa Aderonke Adejuyigbe, Olubukola Adeponle Adesina, Babalola Adeyemi, Salahuddin Ahmed, Francis Akinkunmi, Jalemba Aluvaala, Henry Anyabolu, Shabina Ariff, Sugandha Arya, Ibraheem Awowole, Adejumoke Idowu Ayede, Neelofur Babar, Sumitra Bachani, Rajiv Bahl, Abdullah H. Baqui, Harish Chellani, Saleha Begum Chowdhury, Lynn M. Coppola, Simon Cousens, Pradeep K. Debata, Ayesha de Costa, Sangappa M. Dhaded, Kasturi V. Donimath, Adegoke Gbadegesin Falade, Shivaprasad S. Goudar, Shuchita Gupta, George N. Gwako, Theresa Azonima Irinyenikan, Dennis Anthony Isah, Nigar Jabeen, Arshia Javed, Naima T. Joseph, Rasheda Khanam, John Kinuthia, Oluwafemi Kuti, Tina Lavin, Ahmed R. Laving, Sandhya Maranna, Nicole Minckas, Pratima Mittal, Diwakar Mohan, Sidrah Nausheen, My Huong Nguyen, Olufemi T. Oladapo, Olanike Abosede Olutekunbi, Rosena Olubanke Oluwafemi, Alfred Osoti, Yeshita V. Pujar, Zahida P. Qureshi, Suman P. N. Rao, Sophie Sarrassat, M. A. Shahed, Mohammod Shahidullah, Lumaan Sheikh, Manjunath S. Somannavar, Sajid Soofi, Jyotsna Suri, Sunil S. Vernekar, Joshua P. Vogel, Nitya Wadhwa, Prakash K. Wari, Fred Were, Blair J. Wylie

**Affiliations:** 1 Department of Maternal, Newborn, Child, Adolescent Health, and Ageing, World Health Organization, Geneva, Switzerland; 2 UNDP/UNFPA/UNICEF/WHO/World Bank Special Programme of Research, Development and Research Training in Human Reproduction (HRP), Department of Sexual and Reproductive Health and Research, World Health Organization, Geneva, Switzerland

**Keywords:** Antenatal corticosteroids, Late preterm birth, Dexamethasone, Betamethasone, Low- resource setting

## Abstract

**Background:**

Preterm birth complications are the leading cause of newborn and under-5 mortality. Over 85% of all preterm births occur in the late preterm period, i.e. between 34 and < 37 weeks of gestation. Antenatal corticosteroids (ACS) prevent mortality and respiratory morbidity when administered to women at high risk of an early preterm birth, i.e. < 34 weeks’ gestation. However, the benefits and risks of ACS in the late preterm period are less clear; both guidelines and practices vary between settings. Emerging evidence suggests that the benefits of ACS may be achievable at lower doses than presently used. This trial aims to determine the efficacy and safety of two ACS regimens compared to placebo, when given to women with a high probability of late preterm birth, in hospitals in low-resource countries.

**Methods:**

WHO ACTION III trial is a parallel-group, three-arm, individually randomized, double-blind, placebo-controlled trial of two ACS regimens: dexamethasone phosphate 4 × 6 mg q12h or betamethasone phosphate 4 × 2 mg q 12 h. The trial is being conducted across seven sites in five countries—Bangladesh, India, Kenya, Nigeria, and Pakistan. Eligible women are those with a gestational age between 34 weeks 0 days and 36 weeks 5 days, who have a high probability of preterm birth between 12 h and 7 days (up to 36 weeks 6 days gestation). The primary outcome is a composite of stillbirth or neonatal death within 72 h of birth or use of newborn respiratory support within 72 h of birth or prior to discharge from hospital, whichever is earlier. Secondary outcomes include safety and health utilization measures for both women and newborns. The sample size is 13,500 women.

**Discussion:**

This trial will evaluate the benefits and possible harms of ACS when used in women likely to have a late preterm birth. It will also evaluate a lower-dose ACS regimen based on literature from pharmacokinetic studies. The results of this trial will provide robust critical evidence on the safe and appropriate use of ACS in the late preterm period internationally.

**Trial registration:**

ISRCTN11434567. Registered on 7 June 2021.

**Supplementary Information:**

The online version contains supplementary material available at 10.1186/s13063-024-07941-0.

## Background

### The global burden of preterm birth

Globally, in 2020, an estimated 13.4 million babies were born preterm, with 85% of these births occurring in moderate to late preterm period, i.e. between 32 to < 37 weeks’ gestation [1]. Approximately 65% of all preterm births occurred in South Asian and sub-Saharan African countries [1]. Complications of preterm birth led to the death of nearly 1 million newborns in the same year and are presently the leading cause of death amongst children under 5 years of age [1, 2]. Preterm newborns are at an increased risk of acute- and long-term respiratory, infectious, and neurological complications. Although these risks are substantially higher in infants born at earlier gestations, late preterm infants also experience a significantly higher rate of morbidity, mortality, and adverse neurodevelopment compared to term-born infants [3, 4].

### Antenatal corticosteroids in the late preterm period

Antenatal corticosteroids (ACS) are a key intervention to mitigate the risk of mortality and morbidity resulting from the complications of preterm birth. Evidence from multiple randomized trials largely in high-income settings has shown that when ACS are administered to women with a high probability of preterm birth prior to 34 weeks’ gestation, they can reduce neonatal mortality and respiratory morbidity [5]. More recently, the World Health Organization (WHO) ACTION I trial has conclusively demonstrated similar results even in low-resource settings, when ACS are used in hospitals in accordance with criteria set out in the guidelines for ACS use by the WHO [6]. However, the benefits and risks of ACS in the late preterm period are less clear.

The most recent update of the Cochrane systematic review on ACS efficacy identified seven trials evaluating ACS in the late preterm period [7]. The review found no discernable effect of ACS on perinatal or neonatal mortality amongst studies that enrolled women from 34 weeks 0 days to < 37 weeks gestation, although there was a reduction in the risk of respiratory distress syndrome (RDS) (RR 0.75, 95% CI 0.60, 0.95). A separate systematic review of five ACS trials that enrolled 3844 women from 34 weeks 0 days gestation drew similar conclusions (reduced need for respiratory support, RR = 0.68, 95% CI 0.47–0.98) [8].

While the Cochrane meta-analysis on ACS in the late preterm period included trials from high-, middle-, and low-income countries, a single trial from the United States—the Antenatal Late Preterm Steroid (ALPS Trial) [9]—accounted for more than 75% of the sample size. The ALPS trial reported that compared to placebo, ACS reduced the need for respiratory support amongst neonates (RR 0.80, 95% CI 0.66–0.97) although there was no difference in RDS. It has been suggested that the difference may have been driven by reduction in transient tachypnoea of the newborn (TTN) [9]. The trial also reported an increase in the risk of hypoglycaemia amongst babies in the ACS arm (RR 1.60, 95% CI 1.37–1.87). Smaller subsequent studies in India and Lebanon (a trial with 310 and a prospective cohort with 295 participants respectively) on ACS in the late preterm period have not reported any reductions in rates of RDS, TTN, or neonatal intensive care unit admissions [10, 11]. The WHO ACTION-II trial that randomized 782 women in India between 34 weeks 0 days and 36 weeks 0 days to ACS or placebo did not find differences in benefit or safety outcomes, though this trial was under-powered to reach a clear conclusion [12].

Concerns regarding ACS safety and efficacy in low- and middle-income countries, including during the late preterm period, were raised by the adverse findings of the Antenatal Corticosteroids Trial (ACT) [13]. ACT was a community-based, cluster-randomized implementation trial conducted in six low- and middle-income countries LMICs (Argentina, Guatemala, India, Kenya, Pakistan, and Zambia), which evaluated a complex intervention that aimed to scale-up ACS use. ACT reported significantly higher rates of neonatal death, stillbirth, and possible maternal infection amongst the intervention clusters. These harmful effects appeared concentrated in newborns at and above the 25th birthweight percentile, in whom the relative increase in mortality was 30%. As birthweight was used as a proxy measure for gestational age in this trial, it would suggest that larger, more mature newborns (higher gestational age) were potentially at greater risk of mortality [13].

### Evaluating a lower dose of ACS

A guiding principle of therapeutics is to give the lowest effective dose required to confer benefit, which can help minimize any risks of harm. Despite being used clinically for over 50 years, the optimal ACS dosing regimen is largely unexplored [14]. Dose-ranging studies have not been performed for ACS. Clinically recommended ACS regimens today are largely similar to those used in the original Liggins trial of 1972 [15]. This is particularly relevant for ACS, as steroids have effects on multiple organ systems in the preterm fetus [16]. The clinical use of ACS for fetal lung development remains off-label, though injectable steroid preparations are readily available, are inexpensive, and have been widely used for decades.

Recent animal studies have demonstrated that significantly lower ACS doses than presently used can induce fetal lung maturational changes, suggesting that current ACS regimens in clinical use may expose the fetus to unnecessarily high steroid levels [17, 18], which could mediate some of the adverse effects noted in trials. Recent studies of steroids in non-pregnant [19, 20] and pregnant women [21] have provided more information on the pharmacokinetics of ACS. Taken together, these animal and human studies suggest that a fetal steroid concentration of 1–4 ng/ml could bring about a lung maturational response. Pharmacokinetic modelling of conventionally used ACS regimens suggest that fetal steroid levels are approximately 3–4 times higher [22]. The duration of fetal exposure to ACS at adequate levels is also critical to the maturational response. The ACTION-I trial reported that longer intervals were associated with better newborn outcomes for early preterm newborns, regardless of gestational age at the time of administration [6].

There are also concerns regarding the longer-term effects of in-utero exposure to ACS on neurodevelopment, learning, and behavioural outcomes in children [23, 24]. These concerns and the possibility that lower ACS doses may still confer benefit has resulted in clinical trials being initiated with ACS regimens using lower doses than that currently recommended by WHO [25]. The recent BETADOSE trial in France compared a single injection of betamethasone (half the conventional dose or 11.4 mg) to two injections of betamethasone (full conventional dose or 22.8 mg) for women at risk of preterm birth before 32 weeks of gestation [26]. The trial results indicated that the half-dose was inferior to the full dose on the need for exogenous surfactant in the newborn on an intention-to-treat (RD 2.4%, 95% CI − 0.3 to 5.2) as well as on a per-protocol analysis (RD 2.2%, 95% CI − 0.6 to 5.1). However, it is possible that exposure to prolonged adequate concentrations necessary for fetal lung maturation may not have occurred with the half dose, given the pharmacokinetics of the betamethasone formulation used (Celestone, i.e. betamethasone phosphate and acetate). The ACTION III trial will study the efficacy of a lower dose of ACS (betamethasone phosphate 2 mg q 12 h), which has been selected based on pharmacokinetic modelling to provide the desired sustained fetal exposure (1–4 ng/ml).

### Differences in international clinical recommendations on the use of ACS in the late preterm period

Existing guidelines consistently recommend ACS for women up to 34 weeks gestation [27–30]. There is, however, variation between recommendations on ACS in the late preterm period. The American College of Obstetricians and Gynecologists (ACOG; USA) guidelines recommend that betamethasone can be considered up to 36 weeks 6 days [27], while, the 2019 iteration of the European consensus guideline removed the recommendation on the use of ACS in the late preterm period [30]. The International Federation of Gynaecology and Obstetrics (FIGO) also recommends against routine use of ACS after 34 weeks of gestation [28] (Additional file 1).

There is currently a lack of clarity on the clinical benefits of ACS use in the late preterm period and uncertainty about the potential for harm. The WHO ACTION III trial will provide valuable information to fill this gap, generating critical data for updating clinical guidelines internationally on ACS use in the late preterm period.

## Methods

The trial protocol is reported in line with the Standard Protocol Items: Recommendations for Interventional Trials (SPIRIT) guidelines (Additional file 2) [31].

### Aims and objectives

The aim of this trial is to assess the benefits and possible harms of two regimens of ACS: (i) dexamethasone phosphate 4 × 6 mg q12 h or (ii) betamethasone phosphate 4 × 2 mg q 12 h) compared to placebo, when given to women in the late preterm period (gestational age of 34 weeks 0 days to 36 weeks 5 days) with a high probability of preterm birth. The primary objectives are to compare the effect of each active ACS arm with placebo on a composite outcome of stillbirth, neonatal death, or use of respiratory support within 72 h of life or prior to discharge from hospital, whichever is earlier. The secondary objectives are to compare the effects of each ACS regimen versus placebo on maternal and neonatal safety and healthcare utilization outcomes.

### Trial design

**Fig. 1:**
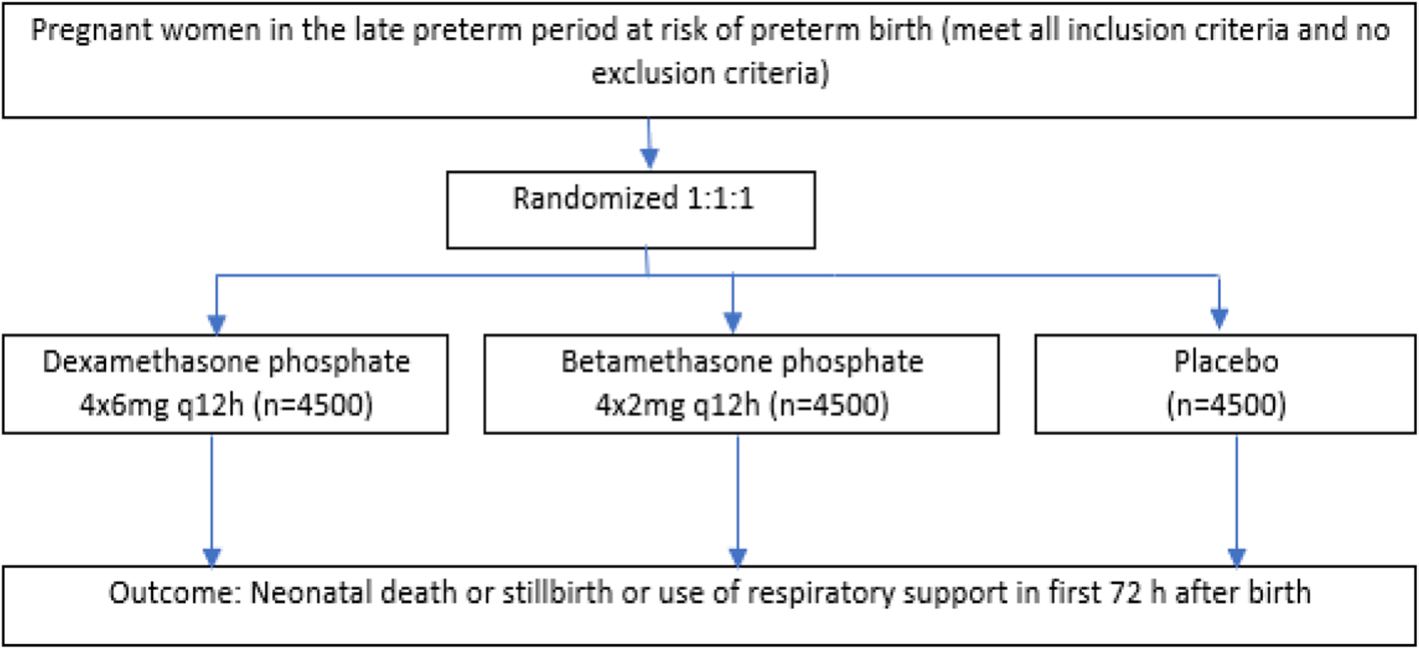
Trial design

ACTION III is a parallel-group, three-arm, individually randomized, double-blind, placebo-controlled trial of two ACS regimens, dexamethasone phosphate 4 × 6 mg q12 h regimen and betamethasone phosphate 4 × 2 mg q 12 h regimen, given to women with a high probability of preterm birth in the late preterm period to improve neonatal outcomes (Fig. 1).

### Study setting

This is a multi-country, multi-centre trial that will be conducted in Bangladesh, India, Kenya, Nigeria, and Pakistan, in hospitals where the WHO ACS treatment criteria can reasonably be met [29]. Specifically, these include hospitals where gestational age assessment can be accurately undertaken, there is high likelihood of preterm birth within 7 days of starting ACS therapy, capacity to recognize and rule out any clinical maternal infection, adequate childbirth care is available (including capacity to recognize and safely manage preterm labour and birth), and the preterm newborn can receive adequate care (including resuscitation, kangaroo mother care, thermal care, feeding support, infection treatment and respiratory support including safe use of oxygen and continuous positive airway pressure [CPAP] as needed) [29].

A total of 29 hospitals will recruit into the trial. The hospitals are largely similar to those that participated in the WHO ACTION I trial [6]. Although these are hospitals with the capacity to manage women having preterm birth and provide care for preterm newborns with minimal out-referral, they do however experience human resource and health system challenges that are common in LMICs. Trial activities will be facility-based, with hospital or community follow-up of recruited women and newborns after hospital discharge to 28 completed days of life.

### Participants

Women with singleton or multiple pregnancy at 34 weeks 0 days to 36 weeks 5 days, with at least one live fetus, and a high probability of late preterm birth will be included. High probability of late preterm birth (up to 36 weeks 6 days) is defined as birth expected between 12 h and 7 days after randomization as a result of one of the following:

- Preterm labour with intact membranes, where preterm labour is defined as at least 6 regular contractions/hour and at least one of the following: cervix ≥ 3 cm dilated or 75% effaced;
- Membranes rupture without preterm labour (preterm labour defined as above;
- Planned delivery by induction of labour or caesarean section between 24 h and 7 days, as deemed necessary by the provider. An induction must be scheduled to start by 36 weeks 5 days at the latest, whereas a caesarean section must be scheduled by 36 weeks 6 days at the latest.

In order to assess eligibility, a good-quality antenatal ultrasound scan with reliable gestational age estimation must be available. If a woman has not received an obstetric ultrasound scan of reasonable quality for gestational age estimation previously (at least two weeks prior to screening) during the current pregnancy, it must be performed as part of eligibility assessment during the screening process.

A woman is ineligible if she is expected to give birth in < 12 h (i.e. if she had ruptured membranes with cervix dilated ≥ 3 cm or effaced ≥ 75%, or with more than 6 contractions per hour or both cervical changes and contractions as specified; or cervical dilation ≥ 8 cm with intact membranes) or if there is evidence of non-reassuring fetal status or other clinical indications that may require immediate preterm delivery. She will be excluded if the obstetric care provider has a clinical suspicion or evidence of clinical chorioamnionitis or severe infection, if she has received any systemic corticosteroid in the preceding 2 weeks (outside of trial), or if no prior ultrasound assessment of gestational age is available and an immediate ultrasound examination is not possible. Other reasons a woman may be ineligible to participate include a major or lethal congenital fetal anomaly being identified, confirmed COVID infection deemed severe enough to require steroid treatment as per national standards of COVID treatment, if the woman is unwilling or unable to provide consent or assent (including due to active labour), is currently participating in another clinical trial, or has previously participated in any ACTION trial or any other clinical indication where the treating clinician considers corticosteroids to be contraindicated.

### Intervention and control

The intervention regimens are (a) dexamethasone phosphate 4 × 6 mg q12 h or (b) betamethasone phosphate 4 × 2 mg q12 h. A single course of 6 mg intramuscular (IM) dexamethasone phosphate or 2 mg IM betamethasone phosphate will be administered every 12 h, to a total of four doses or until birth occurs, whichever occurs first. The control arm will receive four saline injections of the same volume at the same dosing intervals. Women in all arms will receive the same level of standard clinical care. The research staff will record the date and time of administration of each dose. The intervention will be discontinued if participants ask to withdraw from the study at any time, without any coercion.

### Outcomes

#### Primary outcomes

The primary outcome is a composite of stillbirth or neonatal death within 72 h of birth or use of respiratory support within 72 h of birth or prior to discharge from the hospital, whichever is earlier. Use of respiratory support is defined as any one of the following: (i) use of invasive mechanical ventilation, (ii) continuous use of CPAP for 12 h or more with a FiO_2_ ≥ 0.4 at any time, or (iii) continuous use of supplementary oxygen for 24 h or more with a FiO_2_ ≥ 0.4 at any time.

#### Secondary outcomes (also see Additional file 3)

Newborn mortality and respiratory morbidity outcomes: stillbirth; neonatal death within 72 h, 7 days, and 28 days of birth; resuscitation at birth (i.e. use of positive pressure ventilation for > 1 min at birth); severe respiratory distress within 72 h of birth or prior to discharge from the hospital; use of respiratory support within 72 h of birth or prior to discharge from the hospital (as defined above); death or mechanical ventilation or very high CPAP settings (≥ 8 cm water pressure and ≥ 0.7 FiO_2_) in the first 72 h of birth; and cause-specific mortality.

Newborn safety outcomes: neonatal sepsis in the first 7 days of birth; hypoglycaemia in the first 36 h after birth.

Newborn health service utilization outcomes: admission to neonatal care unit in the first 3 days after birth; duration of birth hospitalization; and any parenteral antibiotic use in the first 7 days after birth.

Maternal safety outcomes: maternal death; possible maternal bacterial infection during hospital admission(s); chorioamnionitis; and postpartum endometritis.

Maternal health service utilization outcomes: duration of hospital stay; any therapeutic antibiotic use; and any antibiotic use.

Additionally, all participating sites will report all adverse events for both the mother and the newborn in line with the Good Clinical Practice (GCP) guidelines of the International Council for Harmonization (ICH) GCP, E6(R2), with causality assessment in line with the WHO-UMC system for standardized case causality assessment. We have a detailed standard operating procedure and case record forms for recording the adverse events (two sets of forms for each adverse event—initial and closure). The adverse events are entered into the database and reported to both the Institutional Review Boards and WHO TCU and are monitored periodically by the DSMB. The statistical analysis plan has a detailed section on the plan for analysing adverse events.

### Participant timeline

**Fig. 2:**
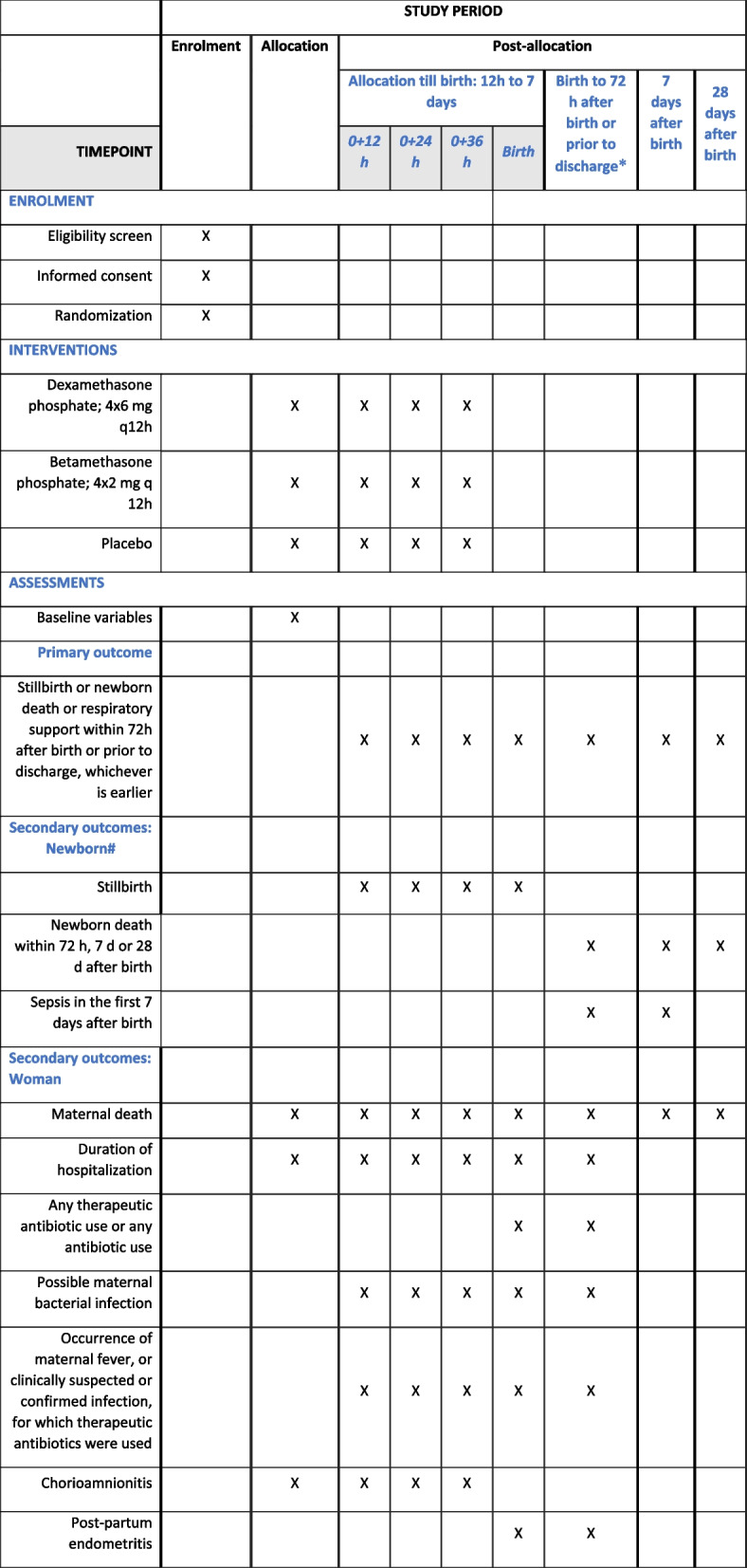
SPIRIT figure. Schedule of enrolment, interventions, and assessments “*” indicates the following: measurement from birth to 72 h after birth or discharge is done every 6 h then every 24 h till day 6 thereafter. “#” indicates the following: other neonatal secondary outcomes include cause-specific mortality; use of positive pressure ventilation > 1 min at birth; hypoglycaemia in first 36 h after birth; newborn severe respiratory distress from birth to 72 h or prior to discharge whichever is earlier; use of newborn respiratory support or high settings for continuous positive airway pressure (CPAP) or mechanical ventilation from birth to 72 h or prior to discharge whichever is earlier; admission to neonatal care unit in first 72 h after birth; and any parenteral antibiotic use till 7 days after birth and duration of birth hospitalization. Another composite secondary newborn outcome is stillbirth OR neonatal death within 72 h after birth OR invasive mechanical ventilation OR need for very high CPAP settings (≥ 8 cm water pressure and ≥ 0.7 FiO_2_) within 72 h after birth or prior to discharge, measured from enrolment till 72 h after birth or discharge, whichever is earlier

The participant timeline and follow-up process are summarized in Fig. 2. Screening, informed consent, and randomization will take place in study hospitals where the mother has presented and will give birth. Randomized women and their newborns will be followed up during the hospital stay and then to 28 days after birth. After randomization, study data will be recorded by trained research staff in participating hospitals. All randomized participants (women and newborns) will have scheduled postpartum/postnatal follow-up visits conducted on day 8 and day 29.

### Screening, informed consent, and recruitment to the trial

Pregnant women admitted to the antenatal, labour ward or emergency admission area in the third trimester at participating hospitals will be routinely evaluated on arrival by obstetric care physicians. Women with clinical features or indications suggestive of preterm birth in the late preterm period will be informed of the study. Study staff (including research or clinical staff trained in study procedures) will conduct formal screening using a standardized screening form. The screening population is women who are between 34 weeks 0 days and 36 weeks 5 days and who are expected to deliver between 12 h and 7 days. Screening will consist of three key activities to assess women for eligibility and trial entry: assessment of gestational age (34 weeks 0 days to 36 weeks 5 days), assessment to ascertain if the delivery is likely between 12 h and 7 days, and assessment for any of the exclusion criteria.

Gestational age will be ascertained based on a best obstetric estimate combining information from the last menstrual period, regularity of cycles, and informed by the earliest ultrasound obtained during the pregnancy. Women will receive an ultrasound assessment for gestational age as part of this screening process if an obstetric ultrasound of reasonable quality for gestational age estimation has not been performed during the current pregnancy at least 2 weeks prior to the screening. In these screening ultrasounds, biometric measurement and gestational age assignment will be performed using INTERGROWTH-21st project biometry guidelines and fetal growth curves, respectively [32] (Additional file 4: Fig. 1). Prior to enrolling patients, all study sites will receive standardized training in the ultrasound evaluation of fetal biometry in the third trimester. Throughout the study, images will be reviewed regularly for quality assurance.

Research staff will seek informed consent from all eligible women who are willing to participate in the trial. All women will receive information about the trial in their language of choice via an information sheet. If willing to participate, the informed consent form will be signed by the participant and study staff. If a potentially eligible woman is a legal minor (according to country’s definitions) and willing to participate in the study, both assent (from the potential participant) and consent (from the parent or legal guardian) will be sought. If any woman is unable to complete the full screening and informed consent process (due to distress, or other reasons), they will not be recruited into the study. Recruitment into the trial will be reviewed by the WHO TCU at monthly intervals. Additional hospitals will be added, and the recruitment period will be extended to meet the target sample size, if required.

### Allocation sequence generation

Participants will be randomly assigned to either dexamethasone-4 × 6 mg, betamethasone-4 × 2 mg ACS regimen, or placebo in a 1:1:1 allocation as per a computer-generated randomization sequence, in balanced permuted blocks. The randomization sequence will be generated by a researcher external to the ACTION III trial. The assignment schedule will be stored at WHO headquarters in Geneva, Switzerland. All sites will receive treatment packs according to the randomization sequence, assembled in special dispensers. Facility study team members at participating sites will remove and open the next pack from the dispenser for allocation as per a set standard operating procedure.

### Allocation concealment and blinding

Allocation concealment will be achieved by having identical treatment packs across the three arms. At the time of randomization, study staff will take the next sequentially numbered pack from the box (Additional file 4: Fig. 2). The IM injections will be administered by the hospital staff nurses according to study procedures.

Participants, care providers, investigators, the WHO trial coordinating unit, data collectors, and the statistical team will be blind to the group allocation. Each active drug will have its own saline placebo identically packaged to allow for blinding of the three arms. Betamethasone phosphate (4 mg/ml as 1 ml ampules) and its placebo (1 ml normal saline) will be manufactured by Recipharm AB®. Dexamethasone (4 mg/ml as 1 ml ampules) and its placebo (1 ml normal saline) will be manufactured by Fresenius Kabi®. To ensure blinding, all three arms will have specially designed identical packaging, appearance, labelling of ampoules, and the same volumes to be administered.

### Emergency unblinding procedures

The principal investigators at each site and a designated WHO project manager will receive the participants’ treatment codes in the form of separate sealed envelopes that contain the treatment allocation for each participant, in case the code for a participant’s treatment needs to be broken urgently.

### Retention and follow-up procedures

The study team will request contact details (address, phone number, relatives) from randomized women, in order to facilitate communication at follow-up to 28 days after birth by home visit. Data collectors will make every reasonable effort to follow the woman and her newborn for the entire study period. After randomization, outcomes occurring in the facility (prior to discharge) will be captured by research study staff working in participating hospitals. At the time of discharge, the woman will be advised to return to the study hospital or call the site investigators in the event of any adverse outcomes for her or her baby.

### Data management

Data will be managed centrally by a data management team, supervised directly by WHO project managers. A web-based, Good Clinical Practice (GCP)-compliant data management platform Kamolo, [Centro Rosarino Estudios Perinatales (CREP)] will be used and be overseen by the site data managers. All data will be collected in study centres on paper case report forms (CRFs). Quality control will be performed at each site, and a validation system will be built into the data entry and management system to ensure consistency, accuracy, and completeness of the data collected. The study statistician will be responsible for the development of the statistical analysis plan and reporting to the DSMB.

### Confidentiality

To ensure participant confidentiality, each participant will be identified by a unique ID number. The local trial register linking personal information and trial ID numbers, and all personal information of participants, will be kept separate from the CRFs. Trial documents will be kept securely under lock and key in the research offices and will not be accessible, other than to the researchers. Data will be entered by trial ID number in the password-protected data management system to which only trial staff will have access. The trial report will not contain the names of any participants, and after completion of the trial, the trial documents will be archived in accordance with institutional and national regulations for clinical research archiving.

## Statistical methods

### Sample size

It is estimated that the prevalence of the primary composite outcome in the control arm will be between 10 and 12% based on data from ACTION I [6], ACTION II [12], and the ALPS trials [9]. A reduction of 20% is the minimal change deemed acceptable in the composite outcome, in order to change practice. Assuming a 2.5% loss to follow-up by 28 days after birth (a conservative estimate based on < 1% loss to follow-up on day 28 in ACTION-I trial in similar sites), a sample size of 4500 women per arm in the three-arm trial will have at least 80% power and α at 0.027 to account for multiple comparisons using Dunnett’s method to detect a 20% reduction in the composite primary outcome.

### Statistical methods for primary and secondary outcomes

The primary intention-to-treat analysis will be based on all participants (i.e. newborns of randomized women, and women) with outcome data available. Data from participants who withdraw their consent for their data to be used will be excluded from the analysis and considered lost to follow-up. For missing data, we will first perform a sensitivity analysis for all those babies for whom we have absolutely no data on and report both best- and worst-case scenarios. The sensitivity analyses will be reported separately. Comparative analyses between trial arms will consider multiplicity as both ACS arms use the same placebo arm as a comparator: confidence intervals for the intervention effect (e.g. risk ratios) will be computed to have a joint 95% coverage probability using Dunnett’s method [33].

The primary outcome and most secondary outcomes are binary variables. For these outcomes, the total number of observations, number of missing values, and percentages will be reported per arm. Comparisons of outcomes between each intervention arm and the placebo arm will be described using risk ratios. Risk ratios will be estimated by binomial generalized estimating equations with log links and robust standard errors to account for potential correlation of outcomes amongst babies born to the same mother. The primary analysis will include arm and site as fixed covariates, and a secondary analysis will also adjust for any baseline covariates for which there is an important imbalance at baseline. If the log binomial models fail to converge, then Poisson models with robust standard errors will be used.

For continuous outcomes (duration of birth hospitalization for the mothers and the babies), the number of participants, missing values, minimum, maximum, means, and standard deviations by arm will be reported. Comparisons of each ACS dose arm against the placebo arm will be described as mean differences. Duration of birth hospitalization (continuous neonatal outcome) for all babies will be compared between arms as mean differences estimated using mixed linear models, including a maternal random effect to account for potential correlation of outcomes amongst babies born to the same mother. The median duration and Kaplan Meier curves will also be reported by arm.

Additionally, maternal and neonatal adverse events that are certainly, probably, or possibly related to the intervention will be shown by trial arm, by site, and overall, in newborns born to randomized women and in randomized women, respectively.

### Interim analyses

A first interim analysis by the data safety monitoring board (DSMB) is tentatively planned at 60% recruitment completion; however, reporting on safety criteria (including adverse events) will occur quarterly. At this first interim analysis, the DSMB will look at the performance of both active arms combined, versus placebo. This is to minimize unnecessary exposure of additional women to the placebo (in case the combined arms are better than placebo). If the analysis reveals that the combined ACS arms are superior to placebo with *p* < 0.001 (Peto’s rule), then the DSMB could recommend cessation of the placebo arm (after confirming that there is statistical evidence that at least one ACS arm is better than placebo with *p* < 0.025, and for safety *p* < 0.01 for mortality. However, recommendations after the results of an interim analysis will be guided not only by statistical considerations but also by practical issues (adverse events, unanticipated costs) as well as clinical considerations or external new information. In the event of both ACS regimens being superior to placebo, and based on a benefit-risk assessment, the DSMB could recommend a non-inferiority comparison between the two active arms.

### Subgroup analyses

Pre- and post-randomization subgroup analyses will be conducted for the primary endpoint. Pre-randomization subgroups include different indications for enrolment (i.e. rupture of membranes, preterm labour with intact membranes, planned termination), gestational age at enrolment (< 34 weeks 6 days, 35 weeks 0 days to 35 weeks 6 days, > 36 weeks 0 days), study site, and single vs multiple births. Post-randomization subgroups include gestational age at birth (preterm (< 37 weeks) vs. not preterm (≥ 37 weeks), interval from time of IMP administration (i.e. first dose) to birth (0 to 12 h, > 12 to 24 h, > 24 h to 7 days, > 7 days), use of tocolytics post-randomization, appropriate size for gestational age (AGA) vs small for gestational age (SGA), and mode of birth (vaginal vs. caesarean section). Statistical tests for effect modification by the different factors mentioned above will be performed.

While post-randomization subgroup analyses are at risk of bias, in the current trial, we believe there are good scientific reasons to investigate these subgroups as there are plausible reasons why the treatment effects could be different. Term newborns (≥ 37 week) exposed to ACS may potentially have different risks compared to those born preterm, as suggested by ACT [13]. The risks of neonatal outcomes have been shown to vary with the ACS administration-to-birth interval [6]. The observed benefits of ACS on neonatal mortality were significantly associated with the use of tocolysis in the ACTION-I trial [6]; this will also be explored in this trial. SGA infants are already exposed to higher levels of endogenous steroids due to pathologic intrauterine stress, and the additional administration of exogenous ACS prior to impending preterm delivery may not offer additional benefit or may even be detrimental [34]. Babies born by caesarean section tend to have higher rates of respiratory morbidity compared to vaginal births as they do not receive the normal physical and hormonal stimuli of passage through the birth canal which enhance fluid reabsorption in fetal lung tissue [35].

Also, these subgroups are clinically important and are explicitly considered in the latest update of WHO ACS recommendations [25]. Before conducting these post-randomization subgroup analyses, we will first examine whether the intervention has an effect on the stratifying variable.

## Trial oversight

Monitoring procedures have been prepared in accordance with the International Council for Harmonisation of Technical Requirements for Pharmaceuticals for Human Use (ICH) harmonized tripartite guideline for Good Clinical Practice E6 (R2). Monitoring activities will be conducted overall, per site, and per hospital. WHO will prepare standard operating procedures for all monitoring activities and will govern all monitoring procedures. Monitoring will be intensive throughout the trial recruitment period and will be conducted by independent trial monitors, principal investigators and co-investigators, and WHO trial coordinating unit (TCU) comprising WHO staff from two WHO Departments (Maternal, Newborn, Child, and Adolescent Health and Ageing, and Sexual and Reproductive Health and Research).

In-person monitoring visits to participating hospitals will be conducted by country investigators, WHO staff, and external, independent clinical trial monitors. These visits will verify that the trial is being conducted according to the study protocol and manual of operations, including screening and informed consent procedures, storage and use of study intervention, data collection and management, and handling of any adverse events. The trial teams will review per-hospital and per-site rates of recruitment, adverse events, and other key progress indicators on a monthly basis. Day-to-day oversight will be conducted by the trial steering committee comprising TCU and the principal investigators at each site. A technical Trial Advisory Group (external independent scientists with expertise in the area of preterm birth) led by an independent chair will advise the trial steering committee.

A study DSMB will comprise five members, including an independent chair, a statistician, and three technical experts familiar with the intervention, maternal and newborn health care, and clinical trial methodology. The DSMB will monitor adverse events on an ongoing basis to look for emerging safety risks and advise the trial coordinating unit (TCU) accordingly. The terms of reference for independent clinical trial monitors and DSMB are available on request from the corresponding authors.

## Ethical considerations

The trial protocol was reviewed and approved by the WHO Ethics Review Committee. All participating sites received approval from the relevant institutional scientific and ethical review committees in the respective country (as well as required permissions from the relevant national regulatory authorities) (Additional files 5, 6 and 7). Any modifications to the protocol which may impact the conduct of the study, a potential benefit of the study participants, or may affect their safety, including changes in study objectives, study design, study population, sample sizes, study procedures, or other significant aspects will require a formal amendment to the protocol. Such amendments will be agreed upon by study co-investigators and submitted to WHO ethics review committee and participating institutional ethics review committees prior to implementation. Insurance has been secured for compensating any participants who suffer harm from trial participation. The results of the study will be published as an open-access, peer-reviewed article in a reputable journal.

## Discussion

As the leading cause of neonatal and child mortality and morbidity, preterm birth is a critical global public health priority. Although > 80% of all preterm births occur in the late preterm period, there is still uncertainty on the balance of benefits and risks of using ACS in women at risk of a late preterm birth. This is evident from the divergent recommendations from different international guidelines [27, 28]. ACOG guidelines recommend that ACS can be given to women up to 36 + 6 weeks, while National Institute for Health and Care Excellence (NICE) guidelines recommend that it can be given up to 35 + 6 weeks gestational age if they are at high risk for preterm birth. Royal College of Obstetricians and Gynaecologists (RCOG) recommends ACS in women with imminent preterm birth anticipated from 35 + 0 to 36 + 6 weeks’ gestation after considering the balance of risks and benefits while the Australia and New Zealand clinical practice guidelines recommends ACS at this gestational age only if there is known lung immaturity and preterm birth is planned or expected within the next 7 days. European consensus guidelines, FIGO guidelines, and guidelines by the Obstetrics Society of Obstetricians and Gynaecologists of Canada and Japan do not support routine ACS use in late preterm infants. In WHO latest ACS recommendations, the Guideline Development Group noted that ACTION III trial would provide the necessary evidence to inform future recommendations [25].

To provide additional information to guide future policies, a sub study to ascertain the pharmacokinetics and pharmacodynamics of ACS is currently being planned. A separate follow-up study is also planned to provide additional information on the longer-term effect of ACS on neurodevelopmental outcomes in early childhood. For all future publications, the authorship will be assigned in line with ICMJE criteria for authorship, as has been done for the current protocol.

The optimal dose of ACS that can confer benefit while minimizing unnecessary fetal exposure has not been studied extensively. This is particularly important, as ACS are a potent developmental modulator, and in utero exposure (particularly for infants born in the late preterm or term period) has been associated with potential neurodevelopmental delays in childhood in retrospective cohort studies [23, 24]. There have been recent calls for trials on lower doses of steroids [36]. The BETADOSE trial in France was the first trial of a single half-dose versus full-dose betamethasone [26]. The betamethasone 4 × 2 mg regimen planned in the ACTION III trial takes into consideration the pharmacokinetics of ACS and the need for a sustained exposure to an adequate concentration over a longer period.

In the ACTION-III trial, we chose a composite outcome of mortality and use of respiratory support as the primary outcome. The DSMB recommended stopping our previous trial to evaluate the safety and efficacy of ACS for late preterm births (ACTION II) prematurely given the lower-than-expected neonatal mortality rate [12]. For the current trial, they recommended using a composite outcome of mortality and respiratory support as biologically, ACS intervention is likely to impact both of these outcomes equally, and clinically, they are both important outcomes related to the complications of prematurity. This composite outcome was agreed upon by external peer reviewers including neonatologists working in this area. As expected, it will also increase statistical efficiency and reduce sample size requirement, costs, and time. This approach has also been used in similar studies like the ALPS trial published in 2016 [9].

The ACTION III trial will also clarify some of the uncertainties raised by ACT [13].

The results of the ACTION III trial will contribute valuable information to bridge the evidence gap on the balance of benefits and risks of ACS in the late preterm period. Given the divergence in guidelines on ACS use in the late preterm period issued by various international and national bodies, in high- and low-income settings, the evidence from this trial will facilitate recommendations on the use of ACS in the late preterm period globally.

## Trial status

This manuscript is based on the current version of the protocol, version 1.16, which was approved by the WHO Ethics Review Committee on 11 April 2023. Recruitment started on 15 July 2022 and is expected to be completed by December 2026.

### Supplementary Information

**Additional file 1.** International recommendations on use of antenatal corticosteroids in the late preterm period.

**Additional file 2.** SPIRIT 2013 checklist: recommended items to address in a clinical trial protocol and related documents.

**Additional file 3.** ACTION-III trial: primary and secondary outcomes.

**Additional file 4.** Supplementary figures.

**Additional file 5.** ACTION III trial informed consent form, v1.4, 2 Dec 2022.

**Additional file 6.** WHO ethics approval.

**Additional file 7.** List of Institutional Review Boards who have approved the ACTION-III trial.

## Data Availability

All relevant materials are available on reasonable request from the corresponding authors.

## Abbreviations

ACS: Antenatal corticosteroids
ACT: Antenatal Corticosteroids Trial
ACTION: Antenatal Corticosteroids for Improving Outcomes in preterm Newborns
DSMB: Data and safety monitoring board
GCP: Good clinical practice
SPIRIT: Standard Protocol Items: Recommendations for Interventional Trials
TCU: Trial coordinating unit
WHO: World Health Organization
