# Supplementary material for: The World Health Organization Antenatal CorTicosteroids for Improving Outcomes in preterm Newborns (ACTION-III) Trial: study protocol for a multi-country, multi-centre, double-blind, three-arm, placebo-controlled, individually randomized trial of antenatal corticosteroids for women at high probability of late preterm birth in hospitals in low- resource countries
Source: Trials. 2024 Apr 12;25:258. doi: 10.1186/s13063-024-07941-0 (PMC11010373; doi:10.1186/s13063-024-07941-0)
Supplement: Supplementary file 1 — Additional file 1. International recommendations on use of antenatal corticosteroids in the late preterm period. [file 13063_2024_7941_MOESM1_ESM.docx]

**Additional file 1. International recommendations on use of antenatal corticosteroids in the late preterm period**

| **Society** | **Gestational age** | **Recommendation** |
| --- | --- | --- |
| American Society of Obstetrics and Gynaecology (ACOG) (US) 2017 (1) | 34+0 to 36+6 weeks | Administration of betamethasone may be considered in pregnant women between 34 0/7 weeks and 36 6/7 weeks of gestation who are at risk of preterm birth within 7 days, and who have not received a previous course of antenatal corticosteroids. |
| NICE Guidelines (England) 2015 (2) | 34+0 to 35+6 weeks | Consider maternal corticosteroids for women between 34+0 and 35+6 weeks of pregnancy who are in suspected, diagnosed or established preterm labour, are having a planned preterm birth, or have P‑PROM. |
| Royal College of Obstetricians and Gynaecologists (RCOG) (UK) (3) | 35+0 to 36+6 weeks’ gestation | Clinicians and women should consider the balance of risks and benefits of corticosteroids in women in whom imminent preterm birth is anticipated from 35+0 to 36+6 weeks’ gestation. |
| Australia and New Zealand’s clinical practice guideline (4) | 35+0 to 36+6 weeks | A single course of corticosteroids should be considered only if there is known lung immaturity  and preterm birth is planned or expected within the next 7 days, even if birth is likely within 24  hours, and regardless of the reason the woman is considered at risk of preterm birth. There is  insufficient evidence on balance of risk/benefit ratio where status of lung immaturity is  unknown (i.e. the vast majority). |
| European consensus guidelines 2022 update (5) | Not recommended after 34 weeks | In women in spontaneous preterm labour after 34 weeks, steroid treatment is controversial and not advisable, as exposure is associated with a significantly higher risk of adverse neurocognitive and psychological outcomes. |
| International Federation of Obstetrics and Gynaecology (6) | Not recommended after 34 weeks | Prenatal corticosteroids should not be offered routinely to women in whom late preterm birth is anticipated. Instead, the use of prenatal corticosteroids should be considered in light of the balance of risks and benefits for individual women. |
| Japan Society of Obstetrics and Gynecology (JSOG) and Japan Association of Obstetricians and Gynecologists (JAOG) (7) | Not recommended after 33 weeks | Administer betamethasone (12 mg twice. i.m., at an interval of 24h) to women if delivery at 22-33 GW is considered to be inevitable. |

**References:**

1. Committee Opinion No. 713 Summary: Antenatal Corticosteroid Therapy for Fetal Maturation. Obstet Gynecol. 2017 Aug;130(2):493-494.
2. Recommendations | Preterm labour and birth | Guidance | NICE. [cited 2023 Aug 30]; Available from: <https://www.nice.org.uk/guidance/ng25/chapter/Recommendations>
3. Stock SJ, Thomson AJ, Papworth S, Royal College of Obstetricians and Gynaecologists. Antenatal corticosteroids to reduce neonatal morbidity and mortality: Green-top Guideline No. 74: Green-top Guideline No. 74. BJOG 2022;129(8):e35–60
4. Antenatal Corticosteroids to Improve Neonatal Outcomes [Internet]. Govt.nz. [cited 2023 Aug 30]. Available from: <https://nationalwomenshealth.adhb.govt.nz/assets/Womens-health/Documents/Policies-and-guidelines/Antenatal-Corticosteroids-to-Improve-Neonatal-Outcomes-3.2017.pdf>
5. Sweet DG, Carnielli V, Greisen G, Hallman M, Ozek E, Te Pas A, et al. European consensus guidelines on the management of respiratory distress syndrome - 2019 update. Neonatology 2019;115(4):432–50. doi: http://dx.doi.org/10.1159/000499361
6. Norman J, Shennan A, Jacobsson B, Stock SJ, FIGO Working Group for Preterm Birth. FIGO good practice recommendations on the use of prenatal corticosteroids to improve outcomes and minimize harm in babies born preterm. Int J Gynaecol Obstet 2021;155(1):26–30. doi: <http://dx.doi.org/10.1002/ijgo.13836>
7. Itakura A, Satoh S, Aoki S, Fukushima K, Hasegawa J, Hyodo H, et al. Guidelines for obstetrical practice in Japan: Japan Society of Obstetrics and Gynecology and Japan Association of Obstetricians and Gynecologists 2020 edition. J Obstet Gynaecol Res 2023;49(1):5–53. Available from: <http://dx.doi.org/10.1111/jog.15438>
