# Supplementary material for: The World Health Organization Antenatal CorTicosteroids for Improving Outcomes in preterm Newborns (ACTION-III) Trial: study protocol for a multi-country, multi-centre, double-blind, three-arm, placebo-controlled, individually randomized trial of antenatal corticosteroids for women at high probability of late preterm birth in hospitals in low- resource countries
Source: Trials. 2024 Apr 12;25:258. doi: 10.1186/s13063-024-07941-0 (PMC11010373; doi:10.1186/s13063-024-07941-0)
Supplement: Supplementary file 3 — Additional file 3. ACTION-III trial: primary and secondary outcomes. [file 13063_2024_7941_MOESM3_ESM.docx]

**ADDITIONAL FILE 3. ACTION-III trial: primary and secondary outcomes**

| **PRIMARY OUTCOMES:** | **OPERATIONAL DEFINITION AND MEASUREMENT** |
| --- | --- |
| Neonatal death <72 hours of birth | Death of a live birth within 72 hours of birth |
| Stillbirth | Any death of a fetus (post-randomization) |
| Respiratory support < 72 hours of birth or prior to discharge from the hospital, whichever is earlier | CPAP for > 12 hours continuously in the first 72 hours after birth with peak FiO2 0.4 (touching 0.4 at any time in the 12 hours) |
|  | Oxygen > 24 hours continuously in first 72 hours after birth with peak FiO2 0.4 (touching 0.4 or it's equivalent at any time in the 24 hours) |
|  | Any invasive mechanical ventilation in first 72 hours after birth |

| **SECONDARY OUTCOMES:** | |
| --- | --- |
| **NEWBORN** | |
| Stillbirth | Any death of a fetus (post-randomization) |
| Neonatal death | Death of a live birth within 28 completed days of birth |
| Early neonatal death | Death of a live birth within 7 completed days of birth |
| Neonatal death <72 hours of birth | Death of a live birth within 72 hours of birth |
| Resuscitation at birth | Use of positive pressure ventilation (PPV) for >1 min (with bag and mask or T-piece) at birth |
| Severe respiratory distress <72 hours of birth or prior to discharge from the hospital, whichever is earlier | Severe respiratory distress (SRD) is defined as any one of the following clinical signs: respiratory rate ≥70/ min, chest indrawing, grunting, or SpO2 <90% present for at least 6 hours |
| Use of respiratory support in first 72 hours of birth or prior to discharge from the hospital, whichever is earlier | Any one of the following in the first 72 hours of birth: (i) invasive mechanical ventilation (ii) continuous use of CPAP for 12 hours or more with an FiO2 ≥0.4 at any time (iii) continuous use of supplementary oxygen for 24 hours or more with an FiO2 ≥0.4 at any time |
| Death or mechanical ventilation or very high CPAP settings in the first 72 hours after birth | Stillbirth OR neonatal death in 72h OR invasive mechanical ventilation in 72h OR need for very high CPAP settings (≥8 cm water pressure and ≥0.7 FiO2) in the first 72 hours after birth |
| Neonatal sepsis in the first 7 days after birth | Defined as the simultaneous presence of at least two (or  more) of the following signs in the first 7 days after birth:   - Stopped feeding well - Severe chest in-drawing - Fever (body temperature of 38°C or greater) - Hypothermia (body temperature less than 35.5°C) - Movement only when stimulated or no movement at all - Convulsions |
| Hypoglycemia in the first 36 hours after birth | Blood glucose <45 mg% at 6, 12, 24, 36 h or anytime ≤36 h after birth based on a test because of clinical suspicion. It could be measured by point of care glucometer or lab value. |
| Admission to a special care unit /ICU in the first 3 days after birth | Any admission in the special newborn care unit in the first 3 days after birth |
| Duration of hospital stay | Total days of hospital stay during the birth hospitalization. |
| Any parenteral antibiotic use up to day 7 after birth | Parenteral antibiotics administered to the newborn up to day 7 after birth. |
| Cause-specific mortality | Ascertained by two physicians with dissenting opinions settled by a third physician. |
| **MATERNAL** | |
| Possible maternal bacterial infection during hospital admission(s) | Women with maternal fever (≥38.0 C) or clinical signs of infection (obstetric or non-obstetric) AND therapeutic antibiotics were used. Assessment must be made by the obstetric care provider. Measured from randomization to 28 days postpartum (during hospital admission and re-admission only, not in the community) |
| Chorioamnionitis | Chorioamnionitis suspected or confirmed. This assessment must be made by an obstetric care physician. Randomization to 28 days postpartum (during postpartum admission(s)) only |
| Postpartum endometritis | Endometritis suspected or confirmed. This assessment must be made by an obstetric care physician. From delivery to 28 days postpartum (during postpartum admission(s) only) |
| Maternal death | Any maternal death in a trial participant, from the time of randomization to 28 completed days postpartum |
| Duration of total maternal hospitalization for birth | Total number of days post-randomization which women are hospitalized for delivery (initial hospitalization for delivery) |
| Any therapeutic antibiotic use | Any use of therapeutic antibiotics from randomization to 28 completed days postpartum |
| Any antibiotic use | Any use of antibiotic (therapeutic or prophylactic) from randomization to 28 completed days postpartum |
