# Supplementary material for: The World Health Organization Antenatal CorTicosteroids for Improving Outcomes in preterm Newborns (ACTION-III) Trial: study protocol for a multi-country, multi-centre, double-blind, three-arm, placebo-controlled, individually randomized trial of antenatal corticosteroids for women at high probability of late preterm birth in hospitals in low- resource countries
Source: Trials. 2024 Apr 12;25:258. doi: 10.1186/s13063-024-07941-0 (PMC11010373; doi:10.1186/s13063-024-07941-0)

**Additional file 4. Supplementary figures**

| **Fig. 1. Screening flow chart for gestational age assessment** | | | | | | | | | | | | |
| --- | --- | --- | --- | --- | --- | --- | --- | --- | --- | --- | --- | --- |
|  | Screen every woman admitted who meet both the following criteria:   1. Gestational age between 34 weeks 0 days to 36 weeks 6 days based on the best obstetric estimate (LMP and USG) 2. She has high likelihood of preterm birth between 12h and 7d from time of randomization | | | | | | | | | | |  |
|  |  |  |  |  |  |  |  |  |  |  |  |  |
|  |  |  |  |  |  |  |  |  |  |  |  |  |
|  |  |  |  |  |  |  |  |  |  |  |  | |
|  |  |  |  |  | Is LMP available, certain and reliable? | | |  |  |  |  | |
|  |  |  |  |  |  |  |  |  |  |  |  | |
|  |  |  |  | Yes |  | No |  | LMP not available |  |  |  | |
|  |  |  |  |  |  |  |  |  |  |  |  | |
|  |  |  |  | Note LMP |  |  |  |  |  |  |  | |
|  |  |  |  |  |  |  |  |  |  |  |  | |
|  |  |  |  | Is reliable first, second or third trimester USG performed at least 2 weeks prior to screening available? | | | | |  |  |  | |
|  |  |  |  |  |  |  |  |  |  |  |  | |
|  |  |  |  | Yes |  |  |  | No |  |  |  | |
|  |  |  |  |  |  |  |  |  |  |  |  | |
|  |  |  |  |  |  |  | Get a third trimester USG done now | | |  |  | |
|  |  |  |  |  |  |  |  |  |  |  |  | |
|  |  |  |  | Corroborate LMP and USG | |  |  |  |  |  |  | |
|  |  |  |  |  |  |  |  |  |  |  |  | |
|  |  |  |  |  |  |  |  |  |  |  |  | |
|  |  |  |  | Is gestational age between 34 weeks 0 days and 36 weeks 5 days* | | | |  | USG cannot be done | |  | |
|  |  |  |  |  |  |  |  |  |  |  |  | |
|  |  |  |  | Yes |  |  | No |  |  |  |  | |
|  |  |  |  |  |  |  |  |  |  |  |  | |
|  |  |  |  | Screen for all eligibility criteria |  |  | Ineligible, end screening |  | Ineligible, end ­­screening |  |  | |
| ­­­ |  |  |  |  |  |  |  |  |  |  |  | |

**Figure 2. Study dispensers and packaging of investigational product**


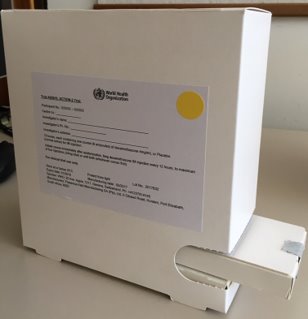


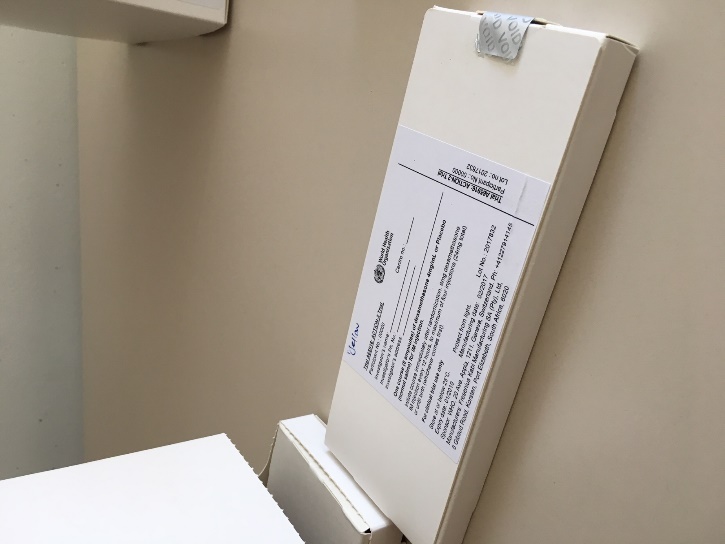


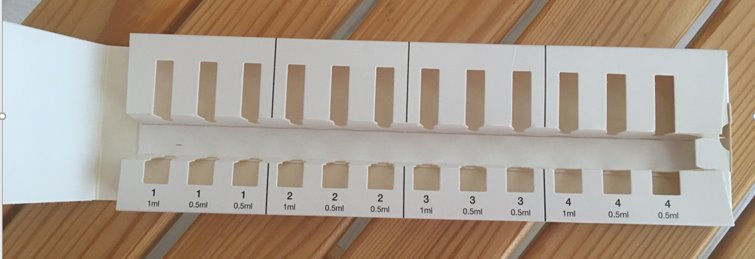

Supplement: Supplementary file 4 — Additional file 4. Supplementary figures. [file 13063_2024_7941_MOESM4_ESM.docx]
