# Supplementary material for: The World Health Organization Antenatal CorTicosteroids for Improving Outcomes in preterm Newborns (ACTION-III) Trial: study protocol for a multi-country, multi-centre, double-blind, three-arm, placebo-controlled, individually randomized trial of antenatal corticosteroids for women at high probability of late preterm birth in hospitals in low- resource countries
Source: Trials. 2024 Apr 12;25:258. doi: 10.1186/s13063-024-07941-0 (PMC11010373; doi:10.1186/s13063-024-07941-0)
Supplement: Supplementary file 5 — Additional file 5. ACTION III trial informed consent form, v1.4, 2 Dec 2022. [file 13063_2024_7941_MOESM5_ESM.docx]

**[Name of Principle Investigator]**

[SITE INSTITUTIONAL LETTERHEAD]

**Informed Consent Form (ICF)**

This Informed Consent Form (ICF) is for women attending participating hospitals in [**COUNTRY**], and who we are inviting to participate in research on use of antenatal corticosteroids for women who are likely to deliver a preterm baby later in pregnancy, for example, at the stage of pregnancy where you are.

The title of our research project is "(number) ACTION III: A multi-country, multi-centre, three-arm, parallel group, double-blind, placebo-controlled, randomized trial of two doses of antenatal corticosteroids for women with a high probability of birth in the late preterm period in hospitals in low-resource countries to improve newborn outcomes"

**[Name of Principal Investigator]**

**[Name of Organization]**

**SPONSOR: World Health Organization**

**[Name of Proposal and version]**

**This Informed Consent Form has two parts:**

- **Information Sheet (to share information about the research with you)**
- **Certificate of Consent (for signatures if you agree to take part)**

**You will be given a copy of the full Informed Consent Form**

**PART I: Information Sheet**

**Introduction**

Good day, I am […….], working for [……..]. We are doing a research project on preterm birth, which is a common condition affecting pregnant women. I am going to give you information and invite you to be part of this research project. You do not have to decide right now whether or not you will participate in the research. Before you decide, you can talk to anyone you feel comfortable with about the research.

There may be some words that you do not understand. Please ask me to stop as we go through the information and I will take time to explain. If you have questions, you can ask them to me, the research doctor or the staff.

**Purpose of the research**

Preterm birth (when a baby is born early in pregnancy, for example before 37 weeks) is a common condition. It affects around 10% of pregnancies. When a woman is likely to give birth too early in pregnancy, it is common practice for the doctors to give her an injection of steroids. This injection can speed up the development of the baby’s lungs, so that when they are born, they have a better chance of survival. The injection can also prevent or reduce other illnesses that affect very preterm babies.

However, the benefits of steroid injection given to women who are likely to deliver a preterm baby later in pregnancy, for example, at the stage of pregnancy at which you are, are not clear. One big study from high income setting suggests that the steroid injections do benefit the baby even at this stage of pregnancy, for example, fewer babies born to mothers who received the steroid injection required respiratory support compared to those who did not receive it. However, it is not clear if this benefit is enough to recommend the use of steroids for all women at your stage of pregnancy, especially in low resource settings like ours.

There is still no research that tells us that there is a good basis for a choice between using or not using the steroid injections for women at the stage of pregnancy at which you are. This research will try to establish whether these injections are safe and effective for mothers and preterm babies by comparing them to an injection of sterile salt water. It will also help us decide the correct dose, meaning if 2mg of the drug Betamethasone given every 12hs is as good as 6mg of the drug Dexamethasone given every 12hs.

**Type of Research Intervention**

This research will involve some injections into your muscle, usually in your arm. Up to four injections may be given.

**Participant selection**

We are inviting all pregnant women of any age to participate in this research if they are at risk of giving birth too soon, are at the stage of pregnancy you are and attend this hospital

- ***Questions to ensure understanding:*** *Do you know why we are asking you to take part in this study? Do you know what the study is about?*

**Voluntary Participation**

Your participation in this research is entirely voluntary. It is your choice whether to participate or not. Whether you choose to participate or not, all the services you receive at this hospital will continue and nothing will change.

If you choose not to participate in this research project, you will be offered the treatment that is routinely offered in this hospital. You may change your mind and stop participating, even if you agreed earlier.

- ***Questions to ensure understanding:*** *If you decide not to take part in this research study, do you know what your options are? Do you know that you do not have to take part in this research study, if you do not wish to? Do you have any questions?*

**Information on the Trial Drugs Dexamethasone** **and Betamethasone**

During this research, we are testing two types of steroid drugs: dexamethasone and betamethasone. These drugs are used in humans for many different illnesses and are also commonly used in pregnant women with minimal side effects.

We now want to test these drugs on women who are likely to give birth preterm in this hospital later in pregnancy. Some participants may receive any one drug, dexamethasone or betamethasone, and some may not be given either of these drugs. Instead, they might be given an injection of salt water that is sterile. The salt water injection is not known to cause any side effects.

**Procedures and Protocol**

**A. Unfamiliar Procedures**

Because we do not know if giving 2mg of Betamethasone every 12hs or 6mg of Dexamethasone every 12 hs are better than salt water in this setting, we need to compare the three. To do this, we will divide all women taking part in this research into three groups. The groups are selected by chance, as if by tossing a coin.

Participants in one group will be given the test drug 1, participants in the second group will be given test drug 2 while participants in the third group will be given the salt water. It is important that neither you nor we know which of the three options you are given. When the research is finished, we will compare which of the three has the best results.

The healthcare workers will be looking after you and the other participating women carefully during the study. If we are concerned about what the drug is doing, we will find out which drug you are getting and make any necessary changes to your care. If there is anything you are concerned about or that is bothering you about the research, please talk to me or one of the other researchers.

You will otherwise receive the normal treatment for your condition, according to national guidelines. This means that you will be closely looked after during labor and delivery. It is possible that you and your baby may receive some tests if they are needed. If any tests are performed as part of routine clinical care, such as blood tests, X-rays or other tests, our researchers will record the results in our files.

**B. Description of the Process**

If you agree to participate, you will receive either 6mg dexamethasone every 12hs or 2mg of betamethasone every 12hs or the salt water.

- During your stay in hospital for childbirth, one of our researchers will collect information on your and your baby’s health.
- After you leave hospital, we would like to contact you by phone or a home visit to see how your and your baby’s health are doing.
- If you or your baby become unwell after you go home, you may need to come back to hospital. If so, we would like you to come back to this hospital. We will also collect information on your and your baby’s health during that admission (if it occurs).
- After 7 days and 28 days have passed from birth, we would like to contact you by phone or visit you at your home to ask you some questions about your and your baby’s health.

**Duration**

The research takes place from the moment you decide to participate before childbirth and up to 28 days after the birth. During that time, it will be necessary for us to contact you by phone message or telephone, and for a researcher to visit you after 28 days have passed after birth. Once the last visit is completed, the research is over.

In the future (for example, some years after the birth) researchers may wish to contact you to see how you and your baby are doing. We would also like permission to contact you in the future, so we can follow up on your and your baby’s health.

- ***Questions to ensure understanding:*** *Do you know how many days after birth we would like to visit you at your home? Do you have any other questions? Do you want me to go through the procedures again?*

**Side Effects**

In this trial, two-third of the participants will receive up to four injections of steroids over 36 hours. This drug can very rarely cause side effects. Any side effects are temporary and will end when the treatment is finished. You may experience difficulty sleeping or headache due to the treatment, however these symptoms can occur often during pregnancy and childbirth. The injection can cause some temporary swelling or soreness around the place where the injection goes into your arm.

While steroids are given to help speed up the development of the baby’s lungs, in some instances, it may cause some side effects for the baby when they are born. They might experience an infection, or low blood sugar. We will follow you and your baby closely and keep track of any problems. We may use some other medicines to decrease the symptoms of the side effects or reactions, or we may stop the use of one or more medicines. If this is necessary, we will discuss it together with you and you will always be consulted before we move to the next step.

**Risks**

Being born preterm increases the risk that your baby will have illnesses in early life, and is at increased risk of death. Participating in this research study will not increase this risk. However, there is a risk that your baby’s health will not be improved by the drug we are testing (i.e. that the drug is not effective).

While the risk of these occurring is low, you should be aware of the possibility. We will try to decrease the chances of this event occurring, but if something unexpected happens, we will ensure you are informed. In the event of harm occurring as a direct result of a study medication(s) or procedure(s) that were used in this study, we will arrange appropriate treatment and you will receive the best available care.

- ***Questions to ensure understanding:*** *Do you understand that you may have some unwanted side-effects from the medicines? Do you understand that participating in this study may carry some risks? Do you have any other questions?*

**Benefits**

There may or may not be any direct benefit for you or your baby in this research. Your participation is likely to help us find the answer to whether these drugs are effective or not and the most effective dose. If the medicine is proven to be effective, we aim to help other people like you benefit from it.

**Reimbursements**

You will not be given any other money or gifts to take part in this research. If any participant (woman or her baby) become ill or are injured as a result of being in this study, they will be given immediate treatment for the illness or injuries at [**HOSPITAL**] according to the hospital’s / Ministry of Health’s guidelines, at no cost.

**Insurance**

All participants in this trial are covered by a clinical trial insurance policy, in the event of harm occurring as a direct result of a study medication(s) or procedure(s) that were used in this study.

- ***Questions to ensure understanding:*** *Can you tell me if you have understood correctly the benefits that you will have if you take part in the study? Do you know if the study will pay you for participating or not? Do you have any other questions?*

**Confidentiality**

It is possible for people in the community to ask question if they become aware that you are participating in this research. However, we will not be sharing your identity or the identity of any women and babies participating in the research. The information that we collect from this research project will be kept confidential. Information about you or your baby that will be collected during the research will be put away, and no-one but the researchers will be able to see it.

Any information about you will have a number on it instead of your name. Only the researchers will know what your number is and we will lock that information up with a lock and key. It will not be shared with or given to anyone else, except researchers at the World Health Organization.

- ***Questions to ensure understanding:*** *Did you understand the procedures that we will be using to make sure that any information that we as researchers collect about you will remain confidential? Do you have any questions about them?*

**Sharing the Results**

Your confidential information will not be shared. Once the research is completed, we will publish the results in order that other interested people may learn from our research and apply what we have learned. We will also make the data from this research available to other researchers, so more can be learnt about these drugs and their optimal dose for this condition. All information about you will be anonymous – it will not contain your name or any personal identifying information.

**Right to Refuse or Withdraw**

You do not have to take part in this research if you do not wish to do so. Refusing to participate will not affect your treatment at this hospital in any way. You may stop participating in the research at any time that you wish without losing any of your rights as a patient here. If you decide to withdraw after receiving some but not all the four doses, you will be given the choice to receive the remaining doses of the assigned drug.

If you consent to participate and later decide to stop participating in the research because of any reason, we will ask you whether you allow us to use your data collected until the time of stopping, or if you prefer us to delete it. If you would like us to delete it, we will remove all data connected to you except information on any serious negative events you may have experienced while you were in the study.Any decision you make related to the use of your data will not affect any treatment or services you or your baby are receiving or might receive in the future.

**Alternatives to participating, and what care would you get or may be available elsewhere if you do not take part in the study**

If you do not wish to take part in the research, you will still be provided with the established standard treatment available at this hospital. That means you will be able to give birth at this hospital and will receive the same care that other women like you normally receive.

**Who to contact?**

If you have any questions you may ask them now or later, even after the study has started. If you wish to ask questions later, you may contact any of the following:

**[name]**

**[address/telephone number/e-mail]**

**This proposal has been reviewed and approved by [name of the local IRB], which is a committee whose task it is to make sure that research participants are protected from harm. If you wish to find about more about the IRB, contact [name, address, telephone number.]). It has also been reviewed by the Ethics Review Committee of the World Health Organization (WHO), which is funding/sponsoring/supporting the study.**

- ***Questions to ensure understanding:*** *Do you know that you do not have to take part in this study if you do not wish to? Do you know that you can ask me questions later, if you wish to? Do you know that I have given the contact details of the person who can give you more information about the study?*

You can ask me any more questions about any part of the research study, if you wish to. Do you have any questions?

**PART II: Certificate of Consent**

**I have read the foregoing information, or it has been read to me. I have had the opportunity to ask questions about it and any questions that I asked have been answered to my satisfaction. I consent voluntarily to participate as a participant in this research.**

**I also agree to be contacted in the future after this research is completed, for longer-term follow up research.**

**Print Name of Participant__________________**

**Signature of Participant ___________________**

**Date ___________________________**

**Day/month/year**

**If illiterate**

A literate witness must sign (if possible, this person should be selected by the participant and should have no connection to the research team). Participants who are illiterate should include their thumb-print as well.

**I have witnessed the accurate reading of the consent form to the potential participant, and the individual has had the opportunity to ask questions. I confirm that the individual has given consent freely.**

**Witness to Print Name of Participant__________________**

**Witness to print name of witness_____________________ AND Thumb print of participant**

**Signature of witness ______________________**

**Date ________________________**

**Day/month/year**

**Statement by the researcher/person taking consent**

**I have accurately read out the information sheet to the potential participant, and to the best of my ability made sure that the participant understands that the following will be done:**

**1.**

**2.**

**3.**

**I confirm that the participant was given an opportunity to ask questions about the study, and all the questions asked by the participant have been answered correctly and to the best of my ability. I confirm that the individual has not been coerced into giving consent, and the consent has been given freely and voluntarily.**

**A copy of this ICF has been provided to the participant.**

**Print Name of Researcher****/person taking the consent________________________**

**Signature of Researcher /person taking the consent__________________________**

**Date ___________________________**

**Day/month/year**
