# Supplementary material for: The World Health Organization Antenatal CorTicosteroids for Improving Outcomes in preterm Newborns (ACTION-III) Trial: study protocol for a multi-country, multi-centre, double-blind, three-arm, placebo-controlled, individually randomized trial of antenatal corticosteroids for women at high probability of late preterm birth in hospitals in low- resource countries
Source: Trials. 2024 Apr 12;25:258. doi: 10.1186/s13063-024-07941-0 (PMC11010373; doi:10.1186/s13063-024-07941-0)
Supplement: Supplementary file 7 — Additional file 7. List of Institutional Review Boards who have approved the ACTION-III trial. [file 13063_2024_7941_MOESM7_ESM.docx]

**Additional file 7. List of Institutional Review Boards that have approved the ACTION-III trial**

| **Site** | **Institutional Review Board** | **Identification number** | **Date of approval** |
| --- | --- | --- | --- |
| Bangladesh | Johns Hopkins School of Public Health Institutional Review Board | IRB No: 15193 | 29 March 2021 |
|  | Bangladesh Medical Research Council National Research Ethics Committee | BMRC/NREC/2019-2022/136 | 14 March 2021 |
| India^[[1]](#footnote-1)^ | Institutional Ethics Committee, KLE Academy of Higher Education and Research, Belagavi | KAHER/EC/2020-21.D-281120002 | 28 November 2020 |
|  | Ethics Committee, Karnataka Institute of Medical Sciences, Hubballi | KIMS/Ethical/412.2020-21 | 29 Jan 2021 |
|  | Institutional Ethics Committee, JJM Medical College, Davangere | JJMMC-IEC/11/2023 | 20 March 2023 |
|  | Institutional Ethics Committee PGIMER and Capital Hospital, Bhubaneswar | IEC/PGIMER&CH, BBSR/Project/2022-23/32 | 26 April 2023 |
|  | Institutional Ethics Committee, Raichur Institute of Medical Sciences, Raichur | RIMS/IEC/2023-24/01 | 13 July 2023 |
|  | Institute Ethics Committee, VMMC and Safdarjung hospital, New Delhi |  | 23 June 2021 |
|  | Institutional Ethics Committee (Human Research), Translational Health Science and Technology Institute | THS 1.8.1/ (117) | 26 March 2021 |
| Kenya | Kenyatta National Hospital – University of Nairobi Ethics Review Committee | KNH-ERC/A/72 | 25 February 2021 |
| Nigeria (Ibadan) | University of Ibadan/University College Hospital Ethics Committee, Ibadan | UI/EC/20/0547  UI/EC/21/0019  UI/EC/21/0020 | 16 March 2021 |
| Nigeria (Ile-Ife) | Obafemi Awolowo University Teaching Hospitals Complex Ethics and Research Committee | ERC/2021/03/03 | 9 March 2021 |
| Pakistan | Aga Khan University Ethics Review Committee | 2021-5888-16925 | 11 March 2021 |
|  | National Bioethics Committee | Ref: No.4-87/NBC-616/23/57 | 17 July , 2023 |
| United Kingdom | London School of Hygiene and Tropical Medicine Observational/Interventions Research Ethics Committee | 22932 | 30 March 2021 |
| United States | Columbia Research  Human Research Protection Office, Institutional Review Boards | IRB-AAAU7974 | 21 August 2023 |

1. The ACTION-3 Trial was also registered with the Clinical Trial Registry of India: CTRI/2017/04/008326 and CTRI/2021/03/032429 [↑](#footnote-ref-1)
