# Supplementary material for: The World Health Organization Antenatal CorTicosteroids for Improving Outcomes in preterm Newborns (ACTION-III) Trial: study protocol for a multi-country, multi-centre, double-blind, three-arm, placebo-controlled, individually randomized trial of antenatal corticosteroids for women at high probability of late preterm birth in hospitals in low- resource countries
Source: Trials. 2024 Apr 12;25:258. doi: 10.1186/s13063-024-07941-0 (PMC11010373; doi:10.1186/s13063-024-07941-0)
Supplement: Supplementary file 6 — Additional file 6. WHO ethics approval. [file 13063_2024_7941_MOESM6_ESM.pdf]

20, AVENUE APPIA – CH-1211 GENEVA 27 – SWITZERLAND – HTTP://WWW.WHO.INT/ETHICS/REVIEW-COMMITTEE/EN/ – HTTPS://EXTRANET.WHO.INT/ERCWEB/LOGIN.PHP

WHO ERC Review Summary

Protocol ID: ERC.0003488
Country: Multi country study
Protocol Title: ACTION III: A multi-country, multi-centre, three-arm, parallel group, double-blind, placebo-controlled, randomized trial of two doses of antenatal corticosteroids for women with a high probability of birth in the late preterm period in hospitals in low-resource countries to improve newborn outcomes
Version: 1.0 **Dated:** 05/11/2020
WHO Responsible Staff Member: Ayesha De Costa
Responsible Unit: WHO/HQ/FWC/MCA
Meeting Date: 21/01/2021

Dear Dr. Ayesha De Costa,
Please find the review summary of the Protocol "ACTION III: A multi-country, multi-centre, three-arm, parallel group, double-blind, placebo-controlled, randomized trial of two doses of antenatal corticosteroids for women with a high probability of birth in the late preterm period in hospitals in low-resource countries to improve newborn outcomes", which was submitted to the Secretariat on 13/11/2020. This proposal underwent regular review.

The outcome of the review is provided below. When responding, please submit the following:

1. A cover memorandum that addresses your responses, POINT BY POINT, to each of the queries in sections A and B.
   Section C contains Suggestions to improve the proposal but there is no obligation to follow them.

2. An Amended protocol including the responses in bold, highlighted or in track changes. Please ensure that tracking formatting changes is switched off or that all formatting changes have been accepted and that no comments which the team may exchange during the editing are included in the track changes version. The protocol should include all relevant documentation (ICF, study instruments, peer review, etc.) even if already submitted.

Please note that comments in the introductory paragraph are meant for the WHO Responsible Staff Member, though you may decide to share them with the PI.

PLEASE RESPOND TO THIS REVIEW SUMMARY WITHIN A three MONTH PERIOD, OR PROVIDE THE ERC SECRETARIAT A VALID JUSTIFICATION FOR THE DELAY.

The ERC considered this an important study with a high potential social value. It is noted that the engaging the Ministry of Health through principal investigators and WRs of the country offices is planned. Ideally, such engagements would have already occurred as to ensure that the study responds to country needs and receives the necessary support.

It was considered that important aspects related to involvement of communities and consent process are to be further developed. An important concern related to how the results of the study may be interpreted is being conveyed. Please find specific comments below.

A. Amendments (Response and change required)

This section includes queries and comments on your protocol, study instruments or the informed consent form for which the ERC requires your response and where relevant, appropriate amendments to the protocol, study instruments or the informed consent.

1. Protocol
 1.1. Please provide an amended proposal specifying the version number and/or date on each page.

In relation to potential risks and benefits:

1.2. The primary outcome of the trial is a composite of stillbirth, neonatal death, or use of respiratory support for the baby with 72 hours of birth. Respiratory support is defined as any of (i) mechanical ventilation (ii) continuous use of CPAP for 12 hours or more with an FiO2 ≥0.4 at any time (iii) continuous use of supplementary oxygen for 24 hours or more with an FiO2 ≥0.4 at any time (p35/265). Although criteria (ii) and (iii) specify continuous use, data will be recorded 6-hourly, and, e.g. 24 hours of oxygen will be defined as oxygen use recorded on four consecutive occasions (p102/265).

Composite outcomes are appropriate in limited circumstances, which do not seem to apply in the context of the study[1]. It is expected that stillbirth and neonatal death will be rare, so the outcome will be dominated by the requirement for respiratory support. Therefore, although stillbirth and neonatal death are included in the outcome, the trial will not in fact be able to detect even relatively large changes in those events. However, perception is likely to be dominated by the presence of stillbirth and neonatal death as outcomes, this has important ethical implications in terms of how study results will be conveyed. Please address.

1.3. Please explain the strategy that will be followed to engage the communities that will take part in the study.

1.4. Please describe the support that will be provided to mother’s whose baby dies or to families who lose the mother in childbirth.

In relation to the consent process:

1.5. Consent from women aged under 18 will be accepted where it is legally possible. In India there is a reporting obligation if women under the age of 18 are found to be pregnant, so that in India only women aged over 18 are to be enrolled. It seems that in order not to exclude minors, who are at high risk of delivering pre-term babies, an option would be that there is no age restriction. Please address.

1.6. In Nigeria the age of consent is 18 years, therefore, participant assent and parental consent is to be used. The woman will be approached first, and if she is willing to participate “an informed assent process conducted with the minor or adolescent” will take place. If she assents to participate, an informed consent process will be conducted with the parent (or guardian)” (p199/265); if they consent the woman will be asked to sign an assent form. However, it is stated (p200/265) that if the “adolescent assents but the parent /guardian does not then the minor’s decision will prevail”. In this context the value of the parental consent is unclear (Noting that there cannot be cases where the woman does not assent but the parent consents). Please address.

1.7. It is stated that women and adolescents and/or their guardians “will be given time” to reflect on whether or not to participate in the study. Given the outcomes that are to be prevented (maternal and or infant death), potential participants may need some time to think over their decision. Please specify the time that will be given to potential participants.

1.8. The consent process is to occur in a calm and private space. Please explain whether this is feasible in the context of the study.

2. Informed Consent Forms

2.1. The ICF states that “Whether you choose to participate or not, all the services you receive at this hospital will continue and nothing will change” (p231/265) and “Refusing to participate will not affect your treatment at this hospital in any way” (p234/265). However, also on p234/265 it is stated “You are also free to go to another hospital, or be referred to another hospital, for your birth if you wish. At that hospital, the final decision on whether you will receive the steroid injection would be taken by you after discussing with the doctors that are taking care of you”. This seems to indicate that women who do not enroll in the trial cannot be given steroids at the trial hospital even if the treating clinician thinks they are indicated. Please modify as appropriate (i.e. transferring a woman who is already in labour, or who has already agreed to a planned Caesarian section (see p65/265), would be inappropriate).

[1] Where the elements of the composite are pathologically equivalent (e.g, “myocardial infarction *or* emergency coronary revascularisation” or “sudden death *or* resuscitated cardiac arrest”) or ethically significant (e.g, stroke causing death *or* dependency).

2.2. Women who withdraw are offered the opportunity to withdraw data already collected (p234/265). However, this offer cannot apply to Serious Adverse Events, and this should be stated.

2.3. It is not clear what would happen to the treatment of women who withdrew after receiving some doses of study drug but not all four. Please address in the consent document and the protocol.

B. Clarifications (Response required but change may not be required)

This section includes queries on your protocol, study instruments or the informed consent form for which the ERC requires to make changes to your proposal.

1. Protocol

1.1. The possibility that a woman may be discharged after randomization and after receiving the allocated intervention is stated (p80/270). She will be encouraged to return to the same facility. Please state the measures that will be taken to ensure that she is given information on who to contact and how -in case she delivers in a different facility. This seems important form the safety point of view.

1.2. In case the participant delivers at home, a home visit will take place. This could lead to a breach in confidentiality (i.e. there are other members of the household present, research assistants are visible to community members, etc.), thus this needs to be addressed with the woman in advance. Engaging the community during the preparatory phase of the study may be helpful in this regard as well.

1.3. In response to the secretariat, the RO has stated that case report forms (CRFs) will be kept at the respective sites, and that there will be no permanent storage. However, it is noted on page 94/270 that WHO is responsible for the permanent storage of data. There seems to be no justification to permanently keep. Please state the data retention period for all study related data.

1.4. In response to the secretariat it has been clarified that some sites will offer reimbursement for travel. It seems that reimbursement for transport should be offered at all sites. Please clarify.

C. Suggestions

This section consists of suggestions for alternative scientific or technical approaches or methods for conducting the research but which do not raise critical, ethical issues. These are meant to be helpful to investigators and are presented as suggestions for you to consider incorporating into a revised protocol. No response from you is required for any comment in this section. If, however, you do make changes to the protocol as a result of these suggestions, please submit the revised protocol to the ERC.

It is noted that the criteria used for ascertaining use of respiratory support creates noise in the data, reducing the power of the study below that calculated statistically.

Protocol ID: ERC.0003488 **Meeting Date**: 21/01/2021

Based on the above comments, the Committee has the following recommendation(s) for this proposal:

[ ] The proposal is ***Approved as submitted.*** No modifications are required.

[X] The proposal is ***Conditionally Approved; requires amendments and/or clarifications.*** Final approval is contingent upon an adequate response by the Principal Investigator, to the satisfaction of the reviewers or the Chair on behalf of the ERC.

[ ] The proposal is ***Not approved; requires additional information and/or rewriting.*** A revised version of the proposal should be re-submitted by the WHO responsible staff member as a new submission to the ERC for re-review by Committee.

[ ] The proposal is ***Rejected***. The proposal is ethically unacceptable, for the reasons stated above. The Principal Investigator may submit a new proposal that takes into consideration the ethical issues raised by the Committee. If you do not agree with the Committee's assessment, please feel free to submit an appeal to the Chair of the ERC, through the Secretariat.

NOTE: Final Approval of the Proposal is contingent upon submission of the following:

[ ] Local ethics approval(s) [ ] Other relevant documents

The ERC would like to receive a copy of the recommendations of the local ethics committee when available.

IMPORTANT

1. *Any changes to the proposal or to the attachments (informed consent/study instruments etc.) should be approved by ERC before being implemented.*
2. *The approval for this proposal is valid for a period of* ***one year only.***
3. *Please resubmit this proposal for a Continuing Review at least 2 months before the next re-approval period.*

Chairperson [signature] **……………………………….** **Date**. 09.02.2021

Name: Peter Olumese

**FINAL APPROVAL**	**FOR THE SECRETARIAT**
Amendments and Clarifications to the proposal have been reviewed. The protocol (Version: 1.13 Date: 19.02.2021) and informed consent Forms (Dated: 15.02.2021) submitted on 31.03.2021 are approved by the ERC.<br><br>Please share the documents approved by the ERC with the local ethics committees.<br><br>**Chairperson** [signature] **……………………………….**<br>**PO/AL**<br>**Name ……………………………….**<br><br>**Date** 13.04.2021 **……………………………….**	Amendments and Clarifications to be reviewed:<br>[ ] Electronically by ERC<br>[ ] by Primary reviewers<br>[ ] by Secretariat<br><br>Local ERC approval(s) obtained on 11.03.2021 Pakistan, 03.03.2021 (Ife Ife) and 16.03.2021 (Ibadan) Nigeria, 25.02.2021 Kenya, 14.03.2021 Bangladesh, 27.11.2020 (Belagavi) and 26.03.2021 (Delhi) India.<br><br>Relevant Documents submitted on: NA
